# CXCL9: evidence and contradictions for its role in tumor progression

**DOI:** 10.1002/cam4.934

**Published:** 2016-10-10

**Authors:** Qiang Ding, Panpan Lu, Yujia Xia, Shuping Ding, Yuhui Fan, Xin Li, Ping Han, Jingmei Liu, Dean Tian, Mei Liu

**Affiliations:** ^1^ Department of GastroenterologyTongji HospitalTongji Medical CollegeHuazhong University of Science and TechnologyWuhanHubei Province430030China

**Keywords:** Cancer, CXCL9, therapy, tumor promoter, tumor suppressor

## Abstract

Chemokines are a group of low molecular weight peptides. Their major function is the recruitment of leukocytes to inflammation sites, but they also play a key role in tumor growth, angiogenesis, and metastasis. In the last few years, accumulated experimental evidence supports that monokine induced by interferon (IFN)‐gamma (CXCL9), a member of CXC chemokine family and known to attract CXCR3‐ (CXCR3‐A and CXCR3‐B) T lymphocytes, is involved in the pathogenesis of a variety of physiologic diseases during their initiation and their maintenance. This review for the first time presents the most comprehensive summary for the role of CXCL9 in different types of tumors, and demonstrates its contradictory role of CXCL9 in tumor progression. Altogether, this is a useful resource for researchers investigating therapeutic opportunities for cancer.

## Introduction

The development of malignant tumors is dependent on the tumor microenvironment, where chemokines and their receptors are important participants 1. Chemokine families are defined as small (8–15 kD) proteins that induce chemotaxis, tissue extravasation and, in some instances, modulate the functional properties of different leukocytes. Chemokines can be subdivided into four classes according to the number and spacing of two conserved N‐terminal cysteine residues, consisting of the C, C‐C, C‐X‐C, and C‐X3‐C families 2. Chemokine CXCL9 is a member of the CXC family and has an important role in the chemotaxis of immune cells. It is secreted by various cell types including immune cells (T lymphocytes, NK cells 3, dendritic cells 4, macrophages 5, eosinophils 6, etc.), and non‐immune cells (hepatic stellate cells 7, preadipocytes, thyrocytes 8, endothelial cell, tumor cells, and fibroblasts 9, etc). CXCL9 has a versatile and controversial role in tumors, and accumulating evidence suggests that CXCL9 is closely associated with the prognosis of tumor patients. Here, the role of CXCL9 in cancer development was reviewed, as well as the molecular mechanisms leading to aberrant expression of CXCL9 in cancer and the potential clinical applications of CXCL9 in diagnosis, prognosis, and cancer treatment.

## Regulation of CXCL9

**Figure 1:**
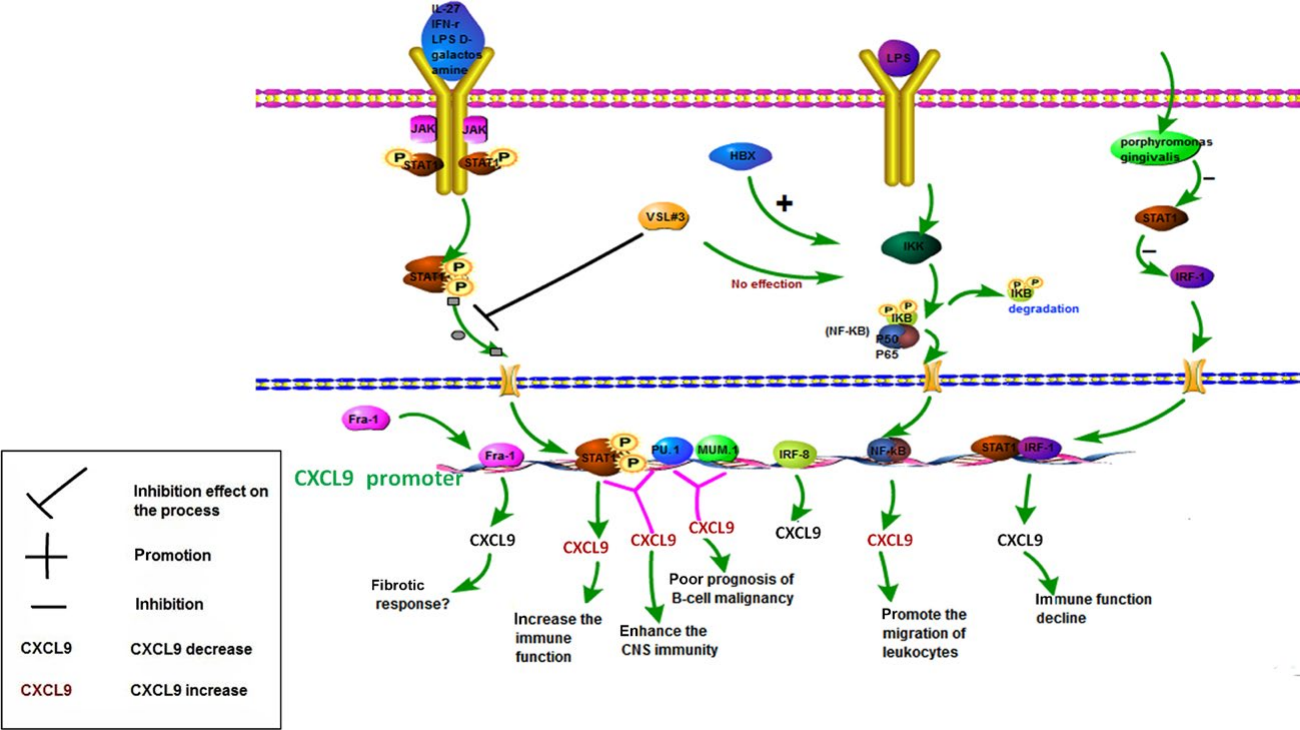
Role and regulation of CXCL9 in cancer. CXCL9 expression could be induced by IFN‐γ, IL‐27, D‐galactosamine, and so on, through JAK/STAT1, PU.1, MUM1, NF‐ kB, and Fra‐1 (direct binding to CXCL9 promoter), and Egr‐1 (not clear). Also, CXCL9 showed a key role on immune function, such as chemotaxis of leukocytes, B‐cells, and T‐cells. STAT1, signal transducer and activator of transcription; JAK, Janus‐activated kinase; PU.1, Myeloid Transcription Factor PU.1; MUM1, multiple myeloma oncogene 1; Egr‐1, Early growth response‐1; Fra‐1, Fos‐related antigen 1.

The transcriptional regulation of CXCL9 is a multistep process involving many transcription factors (Fig. 1), of which signal transducer and activator of transcription (STAT1) and nuclear factor κB (NF‐κB) are two most well‐characterized members 10. Both the gene mutation of STAT1 11 and the blocking of the Janus‐activated kinase (JAK)/STAT‐1 pathway 12, 13 can reduce CXCL9 expression induced by IFN‐γ. Moreover, CXCL9 expression can be suppressed by reducing the levels of components of the STAT1‐IRF‐1(IRF‐1, Interferon regulatory factor) transcriptional activation pathway by Porphyromonas gingivalis that leads to the immune function decline 14. Lipopolysaccharide (LPS) and D‐galactosamine could induce the phosphorylation of STAT1 and enhance the transcription of CXCL9 leading to the enhancement of liver inflammation, and even liver apoptosis and injury 15. In this process, VSL#3 (a mixture of eight different probiotic bacteria) can specifically reduce the phosphorylation of STAT‐1 15, 16. STAT‐1 can also be activated by IL‐27 accompanied with IFN‐γ. Then CXCL9 induced by STAT‐1 finally affects the liver inflammation 17. In addition, our previous work by Xia et al. identified that HBx protein can induce the CXCL9 transcription by activating NF‐κB that binds to its promoter, and CXCL9 promotes the migration of leukocytes in liver with HBV infection 18. Unlike STAT1, phosphorylation of NF‐κB could not be suppressed by VSL#3 16. STAT1 and NF‐κB can cooperatively regulate the expression of CXCL9, and this transcriptional synergy could cause the enhanced recruitment of RNA polymerase II complex to the promoter via simultaneous interaction of CBP with STAT1 and NF‐κB 10.

Besides STAT1 and NF‐κB, myeloid transcription factor PU.1 (PU.1) is also involved in regulating the CXCL9 gene transcription 19, 20. PU.1 is a cell‐specific nuclear transcription factor and a key transcription activator of the gene encoding CXCL9 in response to IFN‐γ. In microglia of the central nervous system (CNS), both STAT1 and PU.1 constitutively bind to the CXCL9 gene promoter. However, only STAT1 binds to the CXCL9 gene promoter in astrocytes, and this binding can be counteracted by the ectopic expression of PU.1 19. Besides, studies also showed that IRF‐8 can bind to the CXCL9 gene promoter induced by IFN‐γ and this binding was accompanied by decreasing PU.1 binding 20. In the B‐cell lymphoma/leukemia, multiple myeloma oncogene 1 (MUM1) can upregulate the expression of CXCL9 by activating its promoter in cooperation with PU.1, leading to poor prognosis of B‐cell malignancy 21.

Early growth response‐1 (Egr‐1), a zinc‐finger transcription factor, also correlates with the expression of CXCL9 in the invading macrophages and accumulation of NK cells in Lewis lung carcinoma 22. Nevertheless, this phenomenon is not observed for all types of tumors and whether Egr‐1 directly regulates CXCL9 or other Egr family members take part in this process is not clearly understood.

CXCL9 is also associated with human hepatic fibrosis and anti‐fibrosis in mice 23, 24. Fos‐related antigen 1 (Fra‐1) represses CXCL9 expression by direct promoter binding in hepatocytes and affects the fibrotic response to some extent 25.

## Receptor CXCR3

CXCR3, a G protein‐coupled receptor, binds to C‐X‐C motif chemokines including CXCL9, CXCL10, CXCL11, CXCL4, and CXCL4L1 26. CXCR3 is highly expressed on T cells, NK cells, and subsets of B cells, and also on epithelial cells, endothelial cells, fibroblasts etc 27, 28. Increasing evidence shows that the abnormal expression of CXCR3 has a significant impact on immune response, inflammation, tumor development, angiogenesis etc 29, 30, 31, 32. There are three spliced variants of CXCR3 in humans, including CXCR3‐A, CXCR3‐B, and CXCR3‐alt, of which only CXCR3‐alt could bind CXCL11. CXCR3‐A and CXCR3‐B are the primary variants, however, their role in physiological functions resembles a double‐edged sword in. In general, CXCR3‐A enhances cell proliferation, chemotaxis, and metastasis, while CXCR3‐B suppresses cell growth, angiogenesis, migration, and promotes apoptosis 26. CXCR3 regulates several signaling pathways, such as MAPK, phospholipase C, and PI3K 33, 34, 35.

## CXCL9 as a Tumor Suppressor

A summary of the increasing evidence about the effects of CXCL9 on tumor suppression is exhibited in Table 1.

**Table 1:** CXCL9 as a tumor suppressor

Type of cancer	CXCL9 Expression	Source	Sample Number	Prognosis^a^	Ref
NSCLC	Low	Tumor cells	109	No relation	38
	High	Tumor cells	12	Might be good	37
	High	Tumor cells	90	Good	36
Breast cancer	High	Tumor cells	60	Good	42
	High	Tumor cells	1058	Good	43
CTCL	High (early), low (advanced)	Tumor cells	9 (early), 13 (advanced)	Might be good	49
	High	Tumor cells	11	Might be good	48
Colorectal cancer	Low	Tumor cells	196	Poor	55
	High	Tumor cells	130	Good	54
UC‐Ca	High	Serum	10	Might be good	56
Melanoma	High	Tumor cells	113	Might be good	60
	High	Tumor cells	44	Might be good	61
Ovarian cancer	High	TCs, macrophages	85	Might be good	66
GC(lymphocyte‐rich)	High	stromal cells, a few TCs	42	Might be good	68
GC	High	mononuclear cells	22	Might be good	69
Ewing sarcoma	High	tumor and stromal cells	20	No relation	70
Cutaneous tumor	High	Tumor cells	42	Might be good	71

NSCLC, Non‐small‐cell lung cancer; CTCL, Cutaneous T‐cell lymphoma; UC‐Ca, ulcerative colitis‐associated cancer; RCC, Renal cell carcinoma; TCs, Tumor cells; GC, Gastric carcinoma.

a The response as “Good” means good prognosis of cancer patients, good response to tumor therapy, or reduction of tumor burden.

### Lung cancer

There is no precise staging system that predicts the prognosis of early‐stage non‐small‐cell lung cancer (NSCLC). Addison et al. found that high protein levels of ELR‐ (Glu‐Leu‐Arg) chemokine CXCL9 existed in 90 human NSCLC tissues. Moreover, they showed that either recombinant human cytokine CXCL9 (rhCXCL9) or gene transfer of CXCL9 inhibited tumor‐derived angiogenesis, suppressing tumor growth and metastasis, which counteracted the angiogenic role of ELR+ chemokine (such as IL‐8 and epithelial neutrophil activating protein 78) partly 36. Metodieva et al. also showed a high expression level of CXCL9 in 12 NSCLC patients 37. However, a study by Kowalczuk et al. also showed that CXCL9 expression was low in 109 NSCLC tumor tissues, but it could not influence both overall and disease‐free survival 38. In addition, other studies have also demonstrated that Egr‐1 deficiency 22, IL‐7 39, CCL21 40, and myeloid‐derived suppressor cells depletion 41 reduced tumor burden by upregulating CXCL9 and CXCL10 expression, which played an anti‐angiogenic role and attracted tumor macrophages, CD4 and CD8+ T lymphocytes, and NK cells. Also, IFN‐γ, interleukin‐12 (IL‐12), and granulocyte macrophage colony‐stimulating factor were involved in the reduction of tumor burden caused by CCL21 40.

### Breast cancer

It is recognized that CXCL9 is significantly associated with lymphocytes infiltration and chemotherapy response in human breast cancer (BC) patients 42, 43, 44. Bronger et al. discovered that a predominantly high mRNA expression of CXCL9 was observed in breast cancer cells in 60 BC tissues 42. Denkert et al. also suggested that the high expression levels of T‐cell‐related markers CD3D and CXCL9 caused a significantly increased pathologic complete response rate (pCR) in 1,058 pretherapeutic BC tissues from two neoadjuvant anthracycline/taxane‐based studies 43. Thakur et al. suggested that the recombined human cytokines IFN‐γ, CXCL9, and CXCL10 could decrease myeloid‐derived suppressor cells (MDSC) population, and might suppress MDSC differentiation 45. Walsern et al. found that in a breast cancer murine model, CXCL9‐expressing tumor cells inhibited local tumor growth and lung metastases via host NK cells, and large numbers of CD4+CXCR3+ and CD8+CXCR3+ host T cells 46. However, Fulton et al. found that the capacity of CXCL9 to inhibit local tumor growth was completely abolished by the depletion of T cells but not compromised by the loss of NK cells, for the reason, T cells inhibited the growth of primary and metastatic tumors, while NK cells controlled transiting tumor cells 47.

### Lymphoma

It is reported that CXCL9 plays a significant role in lymphoma because of its chemotaxis on immunocytes. Tensen et al. showed that CXCL9 and CXCL10 mRNA, not IL‐8, were highly expressed in 11 patients with cutaneous T‐cell lymphoma (CTCL), and correlated with increased CD4+ T cells infiltration, not CD8+ T cells 48. CXCL9 was also reported to be significantly elevated in nine early CTCL compared to the normal skin or 13 advanced CTCL skin 49. Przewoznik et al. showed that the overexpression of CXCL9 and CXCL10 revealed a significant correlation with increased NK cells and their migration in late B‐cell lymphoma stages, which were prerequisites for the potential tumor therapy of adoptive NK‐cell transfer 50. In Addition, IL‐12 and Th1‐derived IFN‐γ exerted antitumor effects through the inhibitory effects of endogenous CXCL9 and CXCL10 on tumor vasculature in human Burkitt's lymphoma 51 and in B‐cell lymphoma 52, respectively.

### Colorectal cancer

Colorectal cancer (CRC) is one of the most prevalent tumor types worldwide. Tumor‐infiltrating T cells are crucial for anti‐tumor immunity 53. Wu et al. showed that CXCL9 was highly expressed in 130 patients’ tumor tissues using PCR and IHC testing, and correlated this with clinic‐pathological features, such as tumor metastasis and differentiation. Moreover, high expression level of CXCL9 predicted a better overall survival 54. Mlecnik et al. found that high expression of CX3CL1, CXCL9, and CXCL10 were correlated with significantly high density of CD8+ T cells, while CXCL9 and CXCL10 attracted memory CD8+ T cells and macrophages, and CX3CL1 attracted effector‐activated cytotoxic T cells and TH1 cells. All of them and the adhesion molecules (ICAM1, VCAM1, MADCAM1) were associated with prolonged disease‐free survival (DFS) 53. Chaput et al. showed that CXCL9 was significantly decreased in tumor tissues from a tissue microarray, consisted of 196 consecutive patients with stage II‐III CRC, which indicated worse relapse‐free survival 55. Watanabe et al. showed that CXCL9 expression was higher in ulcerative colitis‐associated colorectal cancer (UC‐Ca) of 10 patients than in UC‐NonCa of 43 patients, which would improve the accuracy of UC‐Ca diagnosis (positive value 83%, negative value 100%) when combined with 19 other cancer‐related genes, such as cytochrome P450, family 27 B1 (CYP27B1), and Runt‐related transcription factor 3 (RUNX3) 56. A study conducted by Akeus et al. demonstrated endogenous CXCL9 and CXCL10 were selectively increased, followed by accumulation of CXCR3+ conventional T cells, in Treg‐cell‐depleted tumors, which indicated that targeting Treg cells and upregulating CXCL9 and CXCL10 might be a potential immunotherapy 57.

### Melanoma

Tumor‐infiltrating T lymphocytes represent improved prognosis in primary 58 and metastatic melanomas 59. Bedognetti et al. found that CXCL9, CXCL10, CXCL11, and CCL5 were all significantly associated with overall response to therapy in 142 metastatic melanoma patients 60. Moreover, Harlin et al. found that in 44 biopsies of melanoma, highly expressed chemokines, including CXCL9, CCL2, CCL3, CCL4, CCL5, and CXCL10, correlated significantly with CD8+ T‐cell recruitment and migration, which predicted good prognosis for cancer patients 61. Using metastatic‐like melanoma model, Clancy‐Thompson et al. observed that endogenous CXCL9 and CXCL10 were correlated with lungs bearing minimal metastasis lesions by accumulation of CD8+ T cells in a CXCR3‐ and host IFN‐γ‐dependent manner, while it can be suppressed, partly, by adenosine signaling in the tumor microenvironment 62. Deng et al. found that endogenous chemokines CXCL9, CXCL16, CCL12, CCL4, and CCL2 could be significantly increased when DNA methyltransferase 3a that promoted melanoma metastasis and growth was depleted in vivo 63.

### Other tumors

In renal cell carcinoma, endogenous CXCL9 was closely implicated in the antitumor effects that were produced by IL‐2 64 and IL‐12 (also producing CXCL10) 65 by inhibiting tumor angiogenesis and infiltration of CD8+ T lymphocytes, respectively. In ovarian cancer, synergistic effect of tumor‐associated IL‐17 and Th17 cells 66, as well as IL‐18‐primed “helper” NK cells 67, induced the production of endogenous CXCL9 and CXCL10 that were directly correlated with tumor‐infiltrating CD8+ T cells. The former also attracted the NK cells, and the latter also induced CCL5 that was involved in the T‐cells infiltration 66. In gastric carcinoma, endogenous CXCL9 could promote antitumor immunity exerted by T cells, and CXCL10, occasionally, also participated in this process 68, 69. In 20 Ewing sarcoma patients, Berghuis reported that CXCL9, CXCL10, and CCL5 that is highly expressed by tumor and stromal cells were correlated positively with accumulated CD8+ T cells. However, CXCL9 was not related to patient survival rate 70. Moreover, CXCL9 was significantly increased in cutaneous tumor patients, and played a critical role in CXCR3+ T cells (including CD4+ and CD8+ T cells) and NK‐mediated tumor immune‐surveillance and suppression 71, 72. Some other IFN‐regulated proteins (MxA, IDO) were also involved in the recruitment 71.

## CXCL9 as a Tumor Promoter

An increasing body of evidences has been demonstrated that CXCL9 acts as a tumor promoter in multiple types of cancer (summarized in Table 2).

**Table 2:** CXCL9 as a tumor promoter HCC

Type of cancer	CXCL9 expression	Source	Sample number	Prognosis^a^	Ref
HCC	High	Epithelial cells	40	Poor	76
Lung cancer	High	Serum	526	Poor	77
	High	Tumor cells	40	No relation	78
BC (HR+)	High	Serum	40	Might be poor	79
	High	Serum	120	Poor	80
FL (chemotherapy)	High	Serum	392	Poor	81
PCNSL	High	Macrophages, pericytes	22	Might be poor	82
LAHS	High	Serum	15	Poor	83
THRLBL	High	Macrophage	8	Poor	84
ENKL	High	Tumor cells	7	Might be poor	85
Melanoma	High	TuECs	29	Might be poor	87
NPC	High	Serum, tumor cells	205 (serum)/86 (tissue)	Poor/No relation	91
OSCC	High	Serum, tumor cells	181 (serum)/50(tissue)	Poor/Poor	92
Cervical cancer	High	Serum	1057	Might be poor	93
CLL	High	Serum	84	Poor	94
Prostate cancer	High	Tumor cells	20	Poor	95
Glioblastoma	High	Tumor cells	44	Might be poor	97
CNS GCTs	High	Tumor cells	103	Might be poor	98

Hepatocellular carcinoma; BC, Breast cancer; FL, Follicular lymphoma; THRLBCL, TAM of T cell/histiocyte‐rich large B cell lymphoma; PCNSL, Primary central nervous system lymphoma; ENKL, Extranodal natural killer/T‐cell lymphoma; LAHS, Lymphoma‐associated hemophagocytic syndrome; TuECs, tumor endothelial cells; NPC, Nasopharyngeal carcinoma; CNS GCTs, primary central nervous system germ cell tumors, OSCC, Oral cavity squamous cell carcinoma; CLL, Chronic lymphocytic leukemia.

a The response as “Good” means good prognosis of cancer patients, good response to tumor therapy, or reduction of tumor burden.

### Hepatocellular carcinoma

Hepatocellular carcinoma (HCC) causes the third most common cancer mortality due to early metastasis and recurrence 73. Ding et al. in our team showed that CXCL9 promoted the migration and invasion abilities of CD133+ liver cancer cells, and its receptor isoform CXCR3‐A upregulated invasion abilities via activation of p‐ERK1/2‐MMP2/MMP9 pathway induced by rhCXCL9 74. Lan et al. in our team also demonstrated that rhCXCL9 enhanced the invasion ability of hepatocellular carcinoma through the upregulation of phosphatidylinositol‐3, 4, 5‐trisphosphate RAC Exchanger 2 (PREX2) 75. In addition, Liu et al. found that there was pronounced expression of CXCL9, CXCL10, and CXCL11, induced by IL‐17, at the HCC invading edge in 40 patient tissues, which were correlated with the recruitment of CXCR3+ B cells. CXCR3+ B cells could trigger the polarization of protumorigenic M2b macrophages in an IgG‐dependent manner and were positively associated with early recurrence in HCC patients 76.

### Lung cancer

Although CXCL9 is considered as an anti‐tumor factor according to some reports, it could also promote the tumor development as follows. Shiels et al. conducted two independent nested case–control studies (the discovery study and the replication study). The results showed that CXCL9 was associated with lung cancer risk in the replication study, and remained associated with it more than 6 years prior to diagnosis in pooled analyses. Their research suggested that CXCL9 has an etiologic role in lung cancer 77. On the contrary, Nakanishi et al. showed that in 40 lung cancer patients, although CXCL9 was highly upregulated in tumor tissues, no significant relationship between CXCL9 expression and DFS or lower risk of postsurgical recurrence was observed 78.

### Breast cancer

Metastasis of breast cancer (BC) remains a challenge for the therapeutic management. Ejaeidi et al. showed that the levels of CXCL9, CXCL10, and CXCL11 were markedly high in 40 HR+ (hormone receptor) metastatic BC patients’ sera when compared to HR—patients and healthy controls. These three chemokines in sera, especially CXCL10, played an important role in BC development through activation of survivin, β‐catenin, mitogen‐activated protein kinase phosphatase 1 (MKP‐1), and matrix metalloproteinase‐1 (MMP‐1) 79. Ruiz‐Garcia et al. found that among 120 BC patients, the serum concentration of CXCL9 was higher in cancer patients compared with normal volunteers, and the difference between ER‐negative BC samples and normal volunteers was statistically significant. However, it was not different between ER‐positive BC and normal volunteers. When a cutoff of 1000 pg/mL for CXCL9 was selected, the sensitivity of ER‐negative BC diagnosis was 27% and specificity was 90%, and the positive predictive value was 76%. In addition, when CXCL9 combined with fibronectin 1 (Fibronectin 1 > 200 pg/mL as positive; 150 < fibronectin 1 < 200 pg/mL and CXCL9 concentrations >1000 pg/mL as positive), the sensitivity for BC diagnosis was 53% and specificity was 97%. The positive predictive value was up to 96% 80.

### Lymphoma

CXCL9 could also be of great importance for enhancing the understanding of metastasis of lymphoma. Mir et al. demonstrated that in 209 follicular lymphoma patients receiving chemotherapy, elevated serum levels of CXCL9 predicted a shorter median event‐free survival (EFS). When a separate study of 183 patients was combined in a meta‐analysis, the results still showed that CXCL9 was still significantly associated with shorter EFS 81. Venetz et al. found that in primary CNS lymphoma, perivascular CXCL9 correlated with CD8+, CD4+, and Foxp3+ T cells infiltration, and formed heterocomplexes with CXCL12 in vivo. Moreover, rhCXCL9 enhanced rhCXCL12‐induced migration of CXCR3+/CXCR4+/CD8+ T cells and malignant B cells in vitro. These results highlighted the importance of regions expressing chemokines in tumor development 82. Maruoka et al. demonstrated that CXCL9 and CXCL10 (5000 pg/mL and 500 pg/mL as the cutoff levels, respectively) were significantly useful for the early diagnosis and therapeutic outcomes of Lymphoma‐associated hemophagocytic syndrome (LAHS). They also could distinguish LAHS from sepsis and, furthermore, severe from moderate/mild LAHS, and B‐cell‐type from T/NK cell‐type LAHS 83. Many other studies have delineated that CXCL9 was probably involved in tumor‐associated macrophages polarization 84, tumor cell motility 85, and the survival of H‐RS cells 86.

### Melanoma

Amatschek et al. found that low concentration of rhCXCL9 was able to induce melanoma cell migration, conversely at high concentration. And rhCXCL9 enhanced the breakdown of endothelial cells monolayer during transendothelial migration of tumor cells 87. Moreover, RhCXCL9 facilitated the intracellular actin polymerization, cell adhesions (phosphorylation of focal adhesion kinase and paxillin), and cell survival. RhCXCL9 could also increase the intracellular calcium concentration, an early biochemical events in response to chemokines. Endogenous CXCL9 and CXCL10, upregulated by complete Freund's adjuvant in draining lymph nodes, promoted the metastasis of tumor cells to the lymph nodes 88. Jehs et al. reported that uveal melanoma cell lines that were cocultured with activated T cells resulted in an upregulation of chemokines in the supernatant, such as CXCL9, CXCL10, CXCL11, CCL2, CCL5, and VEGF. In turn, these cytokines could generate a tumor‐promoting inflammatory microenvironment 89.

### Head and neck cancer

The head and neck cancer (HNC) is the seventh most common malignancy worldwide, of which squamous cell carcinoma (SCC) is the most common type 90. In a research study that included sera from 205 nasopharyngeal carcinoma (NPC) patients and 231 healthy individuals, and 86 NPC tumor samples, Hsin et al. identified that CXCL9 serum concentrations correlated significantly with tumor stages, nodal stages, overall stages, 5‐year overall survival and DFS in NPC patients, as well as EBV DNA load. However, the immunohistochemical results showed no association between CXCL9 overexpression and clinic‐pathological characteristics 91. Chang et al. found that CXCL9 expression was significantly higher in 50 tumor samples than the normal samples, as well as in the sera of 181 oral cavity SCC patients than the 231 healthy individuals. Serum CXCL9 levels were correlated with pT status, pathological overall stages, tumor depths, and positive bone invasion. Moreover, the results also indicated that higher CXCL9 serum levels predicted a poor prognosis of patient's overall survival and DFS 92.

### Other cancers

In cervical cancer, Zhi et al. detected that serum CXCL9 was significantly increased at invasive International Federation of Gynecology and Obstetrics stages II, and III in a total of 1057 women, as compared to the noninvasive stage. This suggested that CXCL9 was involved in the development of cervical cancer 93. In chronic lymphocytic leukemia, Yan et al. reported that high serum levels of chemokines CL1 (CXCL9, CXCL10, CXCL11, CCL3, CCL4, CCL19, IL‐5, IL‐12, and IFN‐γ) correlated with shorter overall survival, suggesting that high levels of CXCL9 might predict a poor prognosis 94. In prostate cancer (PCa), Hu et al. reported that PCa cells could secrete CXCL9 more than the normal cells, and CD4+ T cells recruited by endogeneous CXCL9, consequently, promoted PCa metastasis via modulation of FGF11/miRNA‐541/AR/MMP9 signaling 95. Liu et al. showed that recombinant mouse and human CXCL9 and CXCL10 facilitated proliferation of murine and human gliomaspheres, suggesting that they may promote tumorigenesis 96. In addition, studies also demonstrate that CXCL9 is highly upregulated in gliolastoma 97 and primary pediatric CNS germ cell tumor (germinoma type) 98.

### CXCL9 in cancer therapy

Accumulating evidence indicates that manipulation of the tumor microenvironment, which involves CXCL9, could enhance the therapeutic efficacy of strategies via tumor‐specific T cells (summarized in Table 3).

**Table 3:** CXCL9 in cancer therapy

Type of cancer	Treatment	CXCL9 expression	Prognosis^a^	Target of CXCL9	Ref
Lung cancer	IL‐7/IL‐7Rα‐Fc	Up	Good	M1 macrophages	99
	IL‐7	Up	Good	anti‐angiogenisis	100
	MIG plus cisplatin	Up	Good	see Note 1	101
Breast cancer	COX‐2 deficiency	Up	Good	CD4+Th cells, CD8+CTL	102
	PGE2/ COX inhibitors	Down/Up	Good	NK cells, T cells	42
	CMF	Up	Good	See Note 2	44
	Lapatinib, doxorubicin	Up	Good	CD8+ T cells	104
Melanoma	ATRA, polyI:C	Up	Good	APCs	106
	IL‐2	Up	Good	TILs	60
HNC	IL‐12	Up	Good	CD4+ T not CD8+ T cells	107
	INF‐ α	UP	Good	anti‐angiogenisis	108
CLL	αDC1	Up	Good	NK, NKT, CD8+T cells	109
RCC	IL‐2	Up	Good	anti‐angiogenisis	110
Genital carcinoma	Imiquimod	Up	Good	CD8+ CTL	111
Sarcoma	OX40L‐Fc	Up	Good	type 1 T‐cell	112
Cutaneous melanoma	temozolomide	Up	Good	Growth inhibition	113

ATRA, all‐transretinoic acid; IL‐12, interleukin‐12; COX‐2, cyclooxygenase 2; CTL, cytotoxic T lymphocytes; polyI:C, polyinosinic:polycytidylic acid PGE2; prostaglandin E2; CMF, cyclophosphamide, methotrexate and 5‐fluorouracile; APCs, antigen‐presenting cells; TILs, tumor‐infiltrating lymphocytes; HNC, Head and neck cancer; CLL, Chronic lymphocytic leukaemia; RCC, Renal cell carcinoma; βDC1, tumor‐loaded α‐type 1‐polarized dendritic cells cocktail (IL‐1β/TNF‐α/IFN‐α/IFN‐γ/poly‐I:C); OX40L‐Fc, OX40 ligand–Fc fusion protein; Note1, anti‐angiogenisis, apoptosis, and CTL activity; Note 2, the target of CXCL9 was not found in the article, but CXCL9 could be a predictive factor.

a The response as “Good” means good prognosis of cancer patients, good response to tumor therapy, or reduction of tumor burden.

### Lung cancer

Although inflammatory responses always occurred with the progression of tumor growth and invasion, cancer cells could escape the cytotoxic effects via immune tolerance. Andersson et al. found that IL‐7/IL‐7Rα‐Fc treatment induced M1 macrophages, reduced tumor burden, and prolonged survival time in mice bearing lung cancer. Depletion of endogenous CXCL9, CXCL10, or IFN‐γ abrogated IL‐7/IL‐7Rα‐Fc‐mediated antitumor activity through reduction of T cells infiltration 99. A further evaluation of genetic immunotherapy by Sharma et al. provided evidence that adenovirus vector expressing interleukin (IL)‐7 (DC‐AdIL‐7) reduced the tumor burden via the increase of IFN‐γ and IL‐12 as well as the antiangiogenic endogenous chemokines CXCL10 and CXCL9, and the decrease of immunosuppressive cytokines TGF‐β and VEGF 100. Additionally, Zhang et al. showed that the combination regime of plasmid‐borne CXCL9 gene therapy plus low‐dose cisplatin augmented the antitumor efficacy by enhancing the tumor anti‐angiogenesis and apoptosis or CTL activity. These effects were shown in Lewis lung carcinoma (LL/2c) murine models, as well as in the colon carcinoma (CT26) murine models 101.

### Breast cancer

Several evidences demonstrate the close relationship between CXCL9 and medicine in BC. The cyclooxygenase‐2 (COX‐2) and its pro‐inflammatory products, prostaglandin E2 (PGE2), are strongly implicated in a range of human cancers including BC in a lymphocyte‐dependent manner 102. Markosyan et al. found that compared to wild‐type mice, ErbB2‐transgenic mice, deficient in mammary epithelial cells COX‐2, showed enhanced immune surveillance by recruiting more CD4+ Th cells and CD8+ CTL, which were coincident with intratumoral CXCL9 influx, a key T‐cell chemoattractant 103. Bronger et al. discovered that there existed inverse correlation between endogenous CXCL9 concentration and COX overexpression in BC tissues. CXCL9 and CXCL10 could be inhibited by PGE2, and induced by unselective COX inhibitors. However, the expected increase of CXCL9 was only observed at low concentrations of COX‐2‐specific inhibitor celecoxib, and decreased by high concentrations 42. Specht et al. discovered that CXCL9, and intersectin 2 (ITSN2) in BC tissues were significantly associated with prolonged DFS in 70 patients with cyclophosphamide, methotrexate, and 5‐fluorouracile (CMF)‐chemotherapy. Furthermore, the results by multivariate Cox analysis showed that the CXCL9/ITSN2 or CXCL9/FLJ22028 (Hypothetical protein FLJ22028 (NM_024854)) ratios could be independent predictive factors of DFS 44. Hannesdóttir et al. showed that the lapatinib and doxorubicin enhanced the expression of Stat1‐dependent endogenous chemokines CXCL9, CXCL10, and CXCL11 (importance for attracting CD8+ T cells), and reduced tumor‐associated macrophages (TAMs), consequently augmenting the antitumor immune response 104.

### Melanoma

The incidence of melanoma has increased significantly, but there are no effective treatments yet 105. Szabo et al. reported that combination of all‐transretinoic acid (ATRA) and polyinosinic:polycytidylic acid (polyI:C) in melanoma cell lines upregulated the expression level of IL‐1β, IL‐6, IFN‐β, CXCL10, CXCL9, CXCL8, and CXCL1 more than treatments with either ATRA or polyI:C separately, which was mediated by toll‐like receptor 3 and MDA5. In this study, they found CXCL9 and CXCL10 to attract activated T lymphocytes 106. Bedognetti et al. found that CXCL9, CXCL10, CXCL11, and CCL5 were all highly expressed in metastatic melanoma patients, and this was associated with responsiveness to adoptive therapy and IL‐2 treatment 60.

### Head and neck cancer

IL‐12 has been known as an effective cytokine for cancer treatment. Using the SCCVII tumor model, Li et al. found that intratumoral and intramuscular injection of IL‐12 gene by electroporation upregulated CXCL9 expression by 15‐fold and 6‐fold, respectively. They also detected that CXCL9 gene injection significantly promoted the infiltration of CD4+ T cells in the tumors, not CD8+ T cells, indicating that CXCL9 played a crucial role in the IL‐12 antitumor efficacy 107. Dorsey et al. showed that intratumoral IFN‐α DNA electroporation caused 50% of tumor eradication rate and more than doubled the survival time when compared with the controls, of which upregulated CXCL9 and CXCL10 had a pivotal role through inhibiting angiogenesis 108.

### Other cancers

In chronic lymphocytic leukemia, Gustafsson et al. detected that tumor‐loaded α‐type 1‐polarized dendritic cells cocktail (IL‐1β/TNF‐α/IFN‐α/IFN‐γ/poly‐I:C;αDC1) showed a better tumor therapeutic efficiency by producing more NK cell‐, NKT cell, and CD8 +  T cell‐recruiting chemokines CXCL9, CXCL10, and CXCL11 in the culture supernatants, as compared with the “standard” cocktail (IL‐1β/TNF‐α/IL‐6/PGE2;PGE2DCs), indicating the key role of the three chemokines in cancer treatment 109. In renal cell carcinoma (RCC), it was reported that high‐dose IL‐2 treatment in 20 patients elevated the plasma levels of CXCR3 ligands (CXCL9, CXCL10, and CXCL11), sequentially, forming an angiostatic environment 110. In external genital carcinoma, Soong et al. found that imiquimod, a toll‐like receptor 7 agonist, induced local expression of CXCL9 and CXCL10 leading to CXCR3+ CD8+ CTL accumulation in the cervicovaginal tract, and enhanced potent antitumor efficacy in the orthotropic cervical cancer model when combined with intramuscular CRT/E7 vaccination 111. In Sarcoma, Pardee et al. identified that OX40 ligand–Fc fusion protein (OX40L‐Fc), a novel tumor necrosis factor receptor family member, promoted anti‐tumor activity in most mice by rendering the tumor microenvironment permissive to type 1 T‐cell infiltration and increase of vascular cell adhesion molecule (VCAM‐1) and CXCL9 expression, upregulated by CD31+ vascular endothelial cells 112. In cutaneous melanoma, Hong et al. discovered that in temozolomide‐treated mice CCL5, CXCL9, and CXCL10 were significantly upregulated, which was followed by T‐cell infiltration, enhanced tumor control, and this prolonged overall survival 113.

### Concluding remarks

Chemokines play a divergent role in controlling the growth and metastasis of malignant tumors. Certain chemokines enhance nonspecific or specific host immunity against tumor implantation, while others could promote tumor growth, metastasis, or neovascularization in tumor tissues. So far, doubts about the role of CXCL9 still exist in tumors, even in the same type of tumor. CXCL9 could promote cancer metastasis via enhanced migration and invasion of tumor cells 74, and breaking of the endothelial cells monolayer 87. However, as a tumor suppressor, it mainly recruited tumor‐infiltrating CD8+ T cells and NK cells 62, and inhibited tumor angiogenesis 36. However, in the section discussing “CXCL9 in cancer therapy” in this review, all the research conducted so far has suggested that high expression level of CXCL9 might be an important target for anti‐cancer therapies. In addition, CXCL9 has been identified as a candidate biomarker in breast cancer 80 and nasopharyngeal carcinoma 91. Therefore, a joint effort among researchers could provide a pave the path to further understand the role of CXCL9 in therapeutic purposes. The complex role of CXCL9 in tumor might be due to the following reasons: First, CXCL9 plays an important role in tumor immunity. CXCL9 could recruit not only CTL, inhibiting tumor development, but also other host immune cells, such as regulatory T cells (Tregs), tumor‐associated macrophages, and MDSC, which mediate immune tolerance in tumors 42, 43, 46, 47. Role of CXCL9 might be dependent on the cancer immune stage, as discussed by Cancer Immunoediting theory that immunity efficacy was different at different stages 114. Second, the contradictory role of CXCL9 might be associated with its receptor's splice variants CXCR3‐A and CXCR3‐B, as they always showed a counteracting role in tumor progression. CXCL9/CXCR3‐A could promote tumor migration and invasion via PI3K 33, MAPK 74, pathways and so on, but CXCL9/CXCR3‐B could inhibit endothelial cells proliferation 115 and tumor angiogenesis 36, which might be mediated by VEGF/VEGFR2 (KDR) and its downstream phospholipase Cγ, p‐JNK, and p‐ERK 24. Lastly, the different functions of CXCL9 were closely related with the many cell types (stated above) secreting it, and its concentration in the tumor microenvironment. CXCL9 will draw more researchers’ interests and attention in the future because of its contradictory and key effects on tumor initiation and development. Nevertheless, a more detailed characterization and mechanism of the role of CXCL9 in tumor biology is desperately required, as it may improve cancer treatment and possibly lead to clinical applications in cancer prognosis, diagnosis, and therapy.

## Conflict of Interest

None declared.
